# Labour pain perception: experiences of Nigerian mothers

**DOI:** 10.11604/pamj.2018.30.288.16672

**Published:** 2018-08-23

**Authors:** Adebayo Adekunle Akadri, Oluwaseyi Isaiah Odelola

**Affiliations:** 1 Department of Obstetrics and Gynaecology, Babcock University, Ilishan-Remo, Ogun State, Nigeria; 2 Department of Obstetrics and Gynaecology, Olabisi Onabanjo University Teaching Hospital, Sagamu, Ogun State, Nigeria

**Keywords:** Analgesia, labour pain, obstetric, perception

## Abstract

**Introduction:**

Labour pain perception is influenced by a variety of factors; hence women experience and cope with labour pain differently. This study was designed to assess labour pain perception among parturient.

**Methods:**

A cross-sectional study involving 132 pregnant women who had vaginal delivery at two tertiary hospitals in south west Nigeria. A structured questionnaire was administered to women within 24 hours of delivery to record details of labour and delivery. Labour pain perception was assessed using the Visual Analogue Score (VAS). Data analysis were done using IBM-SPSS Statistics for Windows version 21.0 (IBM Corp., Armonk, NY, USA).

**Results:**

The mean age of the parturients was 30.6±4.8 years. The mean pain perception of the parturients as assessed by VAS was 7.0 with range of 1.2-10.0. Sixty-six (50%) parturients rated labour pain to be severe (VAS > 7.1). Majority of the respondents 114(86.4%) desired some form of pain relief. The Body Mass Index (BMI) of respondents and birth weight of their babies had statistically significant association with pain perception (p = 0.010; p = 0.038 respectively). Factors associated with increased odds of having severe pain perception include unbooked status, secondary level education, BMI < 30, and gestational age ≥ 37 weeks.

**Conclusion:**

Women in south west Nigeria perceived labour pain as severe and many desired pain relief during labour. Occupation, BMI, gestational age and baby’s birth weight were significant mediating variables in women's experience of labour pain. Modern methods of labour analgesia should be offered to parturients who express desire for it. This will lead to improvements women's childbirth experience.

## Introduction

Labour exposes a woman to one of the most severe forms of pain reported [1]. Despite the severity of labour pain, many women often endure the labour process without any form of analgesia. In some Nigerian cultures, women are more concerned about delivery of a healthy baby than the labour pain [2]. Research suggests that parturients who interpret labour pain as fruitful and purposeful are more likely to feel they can cope [3]. These women are perhaps being motivated by the impending joy associated with delivery of a healthy baby. It is doubtful if women could endure such severe pain under any other condition. Interactions between physiologic, psychosocial and environmental factors influence labour pain perception [4]. Physiological factors such as uterine contractions, cervical dilatation, distension of vagina and perineum affect the intensity of labour pain. Labours that are induced or augmented are often associated with increased pain perception [5, 6]. Reported labour pain is influenced by factors such as maternal age, parity and gestational age at delivery [5, 6]. Psychosocial factors such as culture, ethnicity and educational attainment have been suggested as significant mediating variables in women's experience of labour pain [2, 6-10]. Reported labour pain could also be influenced by fear, anxiety, stress and a sense of abandonment. Environmental factors that may influence labour pain include the type of care providers and the quality of support provided (as perceived by the parturient), the degree of strangeness of the environment, including furniture and equipment, noise, lighting and the restrictiveness of the environment in terms of space or movement with the space [11]. There is no protocol on pain relief in labour in most hospitals in our environment [12]. Apart from occasional use of opioids, the practice commonly involves use of non-pharmacological methods such as labour support, back massage and patterned breathing. A parturient's perception of labour pain is an indication of how she feels she had coped with pain during labour and this is strongly influenced by her socio-cultural background. It is thus inappropriate to extrapolate the level of pain reported by women in one culture to that of another cultural setting [10, 13]. This underscores the importance of ascertaining the perception of labour pain by parturients in a particular cultural setting so as to guide decision on obstetric analgesia services. The aim of this study is to assess parturients' pain perception in two tertiary hospitals in South West Nigeria.

## Methods

This was an observational cross-sectional study. The study was conducted at the obstetric unit of Babcock University Teaching Hospital (BUTH) and Olabisi Onabanjo University Teaching Hospital (OOUTH). BUTH is a faith-based (Seventh-day Adventist) tertiary hospital located in Ilishan-Remo, Ogun state, Nigeria while OOUTH is a State government owned tertiary hospital located in Sagamu, Ogun State, Nigeria. The patients who receive care in these hospitals are of mixed ethnic and socioeconomic background. Ethical approval for the study was obtained from the Babcock University Health Research Ethics Committee (BUHREC). The research was conducted in accordance with the World medical Association Declaration of Helsinki. All study participants were given full information on all aspects of the study and then asked to sign an informed consent form. The study participants were assured of the confidentiality of data obtained from them. The target population for the study was all pregnant women admitted for vaginal delivery at the labour wards of BUTH and OOUTH. The minimum sample size required for the study was estimated using the formula for determining sample size in a descriptive study designed to estimate mean [14].

$$N=\frac{\left(4\sigma^{2}{\left(Z\right)}^{2}\right)}{D^{2}}$$

where N is the sample size; σ is the assumed standard deviation for the group; Z the standard normal deviate set at 1.96 (for 95% confidence interval); D is the total width of the expected confidence interval. In a similar study [6] on perception of labour pain carried out in Enugu, South-East Nigeria, it was found that on a scale of 0 to 10, with 0 representing no pain and 10 representing maximal pain, the mean intensity of pain recorded by the respondents was 7.7 ± 2.8 i.e. σ = 2.8. Assuming the limits of the 95% confidence interval is no more than 0.5 above or 0.5 below the mean intensity of pain score, D = 1. Therefore N = 4 x 2.82 x 1.962 / 1 = 120. Adjustment for a 10% rate of invalid or erroneous entries yielded a final sample size of 132. The inclusion criteria for the study consisted of women who had non-instrumental vaginal delivery at BUTH and OOUTH and gave consent to participate in the study by signing the informed consent form. Women who had instrumental vaginal delivery, women who were delivered by caesarean section, women with associated medical problems like hypertension, diabetes and sickle cell disease, women with pregnancy complications such as abruptio placenta, placenta previa and women who refused to give written informed consent were excluded from the study. Sixty six women were recruited from each of the study centers. These eligible women were recruited consecutively until the estimated sample size was obtained. Women who matched the inclusion criteria were approached within the first 24hrs of delivery and given verbal and written explanation of the study and invited to participate. For those willing to participate, a written informed consent was obtained.

The instrument of data collection was a structured questionnaire which was administered on the subjects within 24hours of delivery. The questionnaire was in two parts. The first part was used to obtain demographic characteristics such as age, occupation, husband's occupation, booking status, religion, tribe, educational attainment, parity, gestational age at delivery, Body Mass Index (BMI). This information was obtained through a review of the parturient's case file. Data on labour characteristics such as its nature of onset, use of oxytocin for augmentation during course of labour, use of episiotomy, foetal birth weight and APGAR score were also recorded. The second part of the questionnaire was used to obtain information on the perception of labour pain. The perceived intensity of labour pain was measured using the visual analogue scale (VAS). The VAS is a 10cm line that is labeled ′no pain′ at one end and ′the worst pain possible′ at the other end. Each subject was asked to indicate on the scale the point corresponding to her perception of the intensity of labour pain. The VAS had been validated as a useful tool in assessment of pain in Nigerian patients [15, 16]. The VAS score was used to categorize pain as either mild (≤ 3.0), moderate (3.1 - 7.0) or severe (7.1 - 10.0) based on a previous definition [17]. The parturient was also asked about her desire for labour pain relief. The data set were anonymized to ensure privacy. Data were analyzed using the IBM-SPSS statistics for Windows version 21.0 (IBM Corp., Armonk, NY, USA). Continuous variables were summarized using descriptive statistics such as mean and standard deviation at 95% confidence interval. Differences in mean pain perception were assessed using independent sample T- test and Analysis of Variance (ANOVA) as appropriate. Categorical variables were summarized using frequencies and percentages. Pearson's Chi-square test was used to establish the association between some respondents' factors and severity of pain perception. Binary logistic regression analysis was used to determine probable predictors of severe pain perception. P-value less than 0.05 was considered statistically significant.

## Results

The mean age of the respondents was 30.6 years (SD = 4.8) with a range of 19 - 44 years. The mean parity was 1.3 (SD = 1.3) with range of 0-7. The mean gestational age at delivery was 38.8 weeks (SD = 1.6). The socio-demographic characteristics of respondents are presented on Table 1. Sixty seven respondents (50.8%) were less than 30 years. Majority of the respondents 104 (78.8%) were of Yoruba ethnicity. Majority of the respondents 79 (59.8%) had tertiary level of education while 7(5.3%) had no formal education. Majority of the respondents were traders and Civil servants accounting for 47 (35.6%) and 45 (34.1%) respectively. Ninety one respondents (68.3%) were Christians while 39 (29.5) were Muslims. There was statistically significant difference in the mean pain scores of respondents with regards to their occupations (F = 2.516, p = 0.033). The mean pain perception of the parturients as assessed by VAS was 7.0 with a range of 1.2-10.0. Sixty-six (50%) of respondents rated labour pain to be severe (VAS > 7.1), 64 (48.5%) rated it moderate (VAS 3.1-7.0) while 2 (1.5%) rated it as mild (VAS ≤ 3.0). Fifty four respondents (40.9%) reported that they perceived the most severe pain during the first stage of labour while 78 (59.1%) reported the most severe pain during active second stage of labour. Majority of the respondents 114 (86.4%) desired some form of pain relief while 18 (13.6%) did not desire any form of pain relief. The association between some respondents' characteristics and pain perception is depicted on Table 2. There was no statistically significant association between booking status, parity, gestational age at delivery and desire for pain relief of respondents, and the severity of pain perception. However there was a statistically significant association between Body Mass Index (BMI) of respondents and pain perception (p = 0.010). Similarly, there was statistically significant association between birth weight and pain perception (p = 0.038).

**Table 1:** Socio-demographic characteristics of respondents

Variable	Frequency	%	Mean Pain Scores
**Age**			
≤30	67	50.8	6.9
>30	65	49.2	7.2
**Ethnicity**			
Yoruba	104	78.8	7.0
Igbo	20	15.2	7.4
Others	8	6.1	6.5
**Occupation**			
Unemployed	9	6.8	5.7
Artisan	18	13.6	6.5
Trader	47	35.6	6.7
Civil Servant	45	34.1	7.7
Professional	4	3.0	8.4
Others	9	6.8	7.1
**Level of education**			
No formal education	7	5.3	6.7
Primary	5	3.8	5.5
Secondary	41	31.1	7.5
Tertiary	79	59.8	6.9
**Religion**			
Christianity	91	68.9	7.2
Islam	39	29.5	6.4
Traditional worship	2	1.5	8.7

**Table 2:** Association between some respondents’ characteristics and pain perception

Variable	Severe Pain perception n(%)	Mild/moderate Pain perception n(%)	N (%)	Chi-square	P-value
**Booking Status**					
Unbooked	18 (62.1)	11 (37.9)	29 (22.0)	2.165	0.207
Booked	48 (46.6)	55 (53.4)	103 (78.0)		
**Parity**					
0	18 (48.6)	19 (51.4)	37 (28.0)	1.900	0.594
1-2	36 (49.3)	37 (50.7)	73 (55.3)		
3-4	8 (47.1)	9 (52.9)	17 (12.9)		
5	4 (80.0)	1 (20.0)	5 (3.8)		
**Body mass Index**					
<18.5	3 (75.0)	1 (25.0)	4 (3.0)	11.267	*0.010
18.5-24.9	29 (48.3)	3 1(51.7)	60 (45.5)		
25.0-29.9	30 (62.5)	18 (37.5)	48 (36.4)		
≥30	4 (20.0)	16 (80.0)	20 (15.2)		
**Gestational age**					
<37	2 (28.6)	5 (71.4)	7 (28.0)	1.358	0.244
≥37	64 (51.2)	61 (48.8)	125 (94.7)		
**Birth weight**					
<2.5	0 (0.0)	6 (100.0)	6 (4.5)	6.519	*0.038
2.5-3.5	55 (53.4)	48 (46.6)	103 (78.0)		
>3.5	11 (47.8)	12 (52.2)	23 (17.4)		
**Desire for pain relief**					
Yes	59 (51.8)	55 (48.2)	114 (86.4)	1.029	0.310
No	7 (38.9)	11 (61.1)	18 (13.6)		

P < 0.05 considered statistically significant

Table 3 shows the logistic regression analysis for some probable predictors of severe pain perception. Unbooked women had increased odds of having severe labour pain perception when compared to booked women (OR=1.9, 95% CI: 0.81- 4.36). Women who had primary and tertiary level of education had reduced odds of having severe labour pain perception when compared to women who had no formal education (OR=0.5, 95% CI: 0.05-5.15; OR = 0.6, CI: 0.12-2.70 respectively). Women who had secondary level education however had increased odds of having severe labour pain (OR=1.3, CI: 0.26-6.61). Women who had parity of 5 and above had increased odds of having severe labour pain perception when compared to those who were nulliparous (OR = 4.2, CI: 0.43- 41.45). Women who were underweight, normal and overweight had increased odds of having severe labour pain perception when compared to obese women (OR = 12.0, CI: 0.97- 142.86; OR = 3.7, CI: 1.12- 12.51; OR = 6.7, CI: 1.93- 23.08 respectively). This pattern was found to be statistically significant in both normal and overweight women (p = 0.03). Women who had term pregnancies had increased odds of having severe labour pain perception when compared to those who had preterm pregnancies (OR = 2.6, CI: 0.49- 14.03).

**Table 3:** Logistic regression analysis for some probable predictors of severe pain perception

Variable	Severe Pain perception n(%)	Mild/moderate Pain perception n(%)	Odds Ratio	95% CI	P value
**Booking Status**					
Unbooked	18 (62.1)	11 (37.9)	1.9	0.81- 4.36	0.14
Booked	48 (46.6)	55 (53.4)	1.0		
**Level of education**					
No formal education	4 (57.1)	3 (42.9)	1.0		
Primary	2 (40.0)	3 (60.0)	0.5	0.05-5.15	0.56
Secondary	26 (63.4)	15 (36.6)	1.3	0.26-6.61	0.75
Tertiary	34 (43.0)	45 (57.0)	0.6	0.12-2.70	0.48
**Parity**					
0	18 (48.6)	19 (51.4)	1.0		
1-2	36 (49.3)	37 (50.7)	1.0	0.47-2.27	0.95
3-4	8 (47.1)	9 (52.9)	0.9	0.30-2.96	0.91
≥5	4 (80.0)	1 (20.0)	4.2	0.43-41.45	0.22
**Body mass Index**					
<18.5 (underweight)	3 (75.0)	1 (25.0)	12.0	0.97-142.86	0.05
18.5-24.9 (Normal)	29 (48.3)	31 (51.7)	3.7	1.12-12.51	*0.03
25.0-29.9 (Overweight)	30 (62.5)	18 (37.5)	6.7	1.93-23.08	*0.03
≥30 (Obese)	4 (20.0)	16 (80.0)	1.0		
**Gestational age**					
<37	2 (28.6)	5 (71.4)	1.0		
≥37	64 (51.2)	61 (48.8)	2.6	0.49-14.03	0.26

P < 0.05 considered statistically significant

## Discussion

Labour pain perception is the parturients' interpretation of the noxious sensory stimuli transmitted during labour. It is a personal experience and has sensory and affective or distress components [18]. The multidimensional nature of labour pain is responsible for the varying ways by which women of different socio-cultural backgrounds experience and cope with pain during labour. This study has shown a substantial proportion of south west Nigerian women perceive labour pain as severe and majority desired effective methods of labour pain relief. Almost all the women in this study rated labour pain as either moderate or severe, with 50% perceiving labour pain as severe. This is similar to findings from a study carried out in south east Nigeria where 52% of women rated labour pain as severe [19]. This study also shows that 86.4% of parturients desired some form of labour pain relief. The same proportion was reported in Ilesa [13], while a similar proportion of 85.1% was reported in Benin [20], in south west and south Nigeria respectively. This finding suggests that the widely propagated notion during antenatal health talk that labour pain must be endured should be discarded [21]. Effective labour pain relief may encourage more women to have hospital deliveries thereby avoiding complications that usually arise when women have their deliveries in unorthodox places [13]. Civil Servants and professionals had a higher mean pain scores when compared to other less westernized occupations like artisans and traders. The pattern was statistically significant. It was also observed that women with secondary and tertiary level of education had higher mean pain perception than those with lower level of education. Similar result was reported in other studies [5, 21]. Findings from this study support the hypothesis that westernization (probably through education) tends to increase reported labour pain [7]. Muslims had lower mean pain scores than Christians. This may be a reflection of religiously or culturally learned values that influence expression of labour pain. These accepted values may suggest that women should go through labour in a stoic manner [9]. This fact should be borne in mind by the health care team so as avoid delays in diagnosis of intrapartum complications like placental abruption and uterine rupture which also present with abdominal pain.

The age and parity of parturients did not have any significant influence on labour pain perception. Our findings confirm the result of other authors in south west Nigeria who also found that labour pain had no significant association with maternal age and parity [13]. Unbooked parturients had increased odds of having severe pain perception when compared to booked parturients. This finding may be because unbooked parturients did not benefit from the antenatal health talks which may have prepared them to better cope with labour pain. Some authors have reported that the women with lower expectation of pain before labour usually experience lower pain during labour [22]. There was no statistical association between gestational age at delivery and maternal labour pain perception. However, women who delivered at term had increased odds of having severe pain perception when compared with women who delivered preterm. The reason for this may be extrapolated from the statistically significant association noted between the birth weight and pain perception. Severe pain was commoner in parturients with average sized babies when compared to those with low birth weight babies. Labour pain during the second stage of labour is usually due to the distention of vagina and perineum. It is likely that the bigger the baby, the more severe the distention and therefore more severe pain. There was a statistically significant association between the body mass index (BMI) of parturients and labour pain perception. Women who were underweight, normal and overweight had increased odds of having severe pain perception when compared to obese women. This finding is however at variance with Klostergaard *et al* [22] where the authors did not find any significant relationship between body mass index and pain perception. This disparity justifies the need for further research.

## Conclusion

This study has shown that many women in south west Nigeria perceive labour pain as severe and a substantial proportion desire pain relief during labour. Occupation, BMI, gestational age and baby’s birth weight were significant mediating variables in women's experience of labour pain. Modern methods of labour analgesia should be offered to parturients who express desire for it. This will lead to a reduction in unmet need for obstetric analgesia and improve women's childbirth experience.

### What is known about this topic

- Labour pain perception is influenced by a variety of factors;
- Women in different socio-cultural settings experience and cope with labour pain differently.

### What this study adds

- Many women in south west Nigeria perceive labour pain as severe and desire labour pain relief;
- Women's occupations and baby's birth weights influence labour pain perception;
- Obese women have lower pain perception than overweight, normal weight or underweight women.

## Competing interests

The authors declare no competing interest.
